# Anatomy, Physiology, and Pathological Anatomy

**Published:** 1837-01

**Authors:** 


### PART FOURTH.

### Selections from the British Journals

(FOR THE QUARTER ENDING NOV. 30, 1836).

---

### ANATOMY, PHYSIOLOGY, AND PATHOLOGICAL ANATOMY.

### *On the Anatomy of the Fifth Nerve.* By B. Alcock, M.B.

This paper was communicated to the British Association at Bristol. It contains a brief account of some particulars respecting the 5th nerve, which the author believes to have been hitherto unobserved. These particulars are the following :—1. Both packets of the nerve can be traced to the same point of attachment, behind the middle crus of the cerebellum, in the angle formed by the three crura, behind and beneath the middle, above the inferior, and before the superior. 2. The point of attachment presents, when the surrounding portion has been pushed away from it, a small eminence which is imbedded in the substance forming the floor of the fourth ventricle, separated from the interior of the chamber by a thin lamina only. 3. Besides the fasciculus discovered by Rolando, Dr. A. has found a second and smaller fasciculus, which descends from the point of attachment, behind and within the inferior crus, and, between the posterior pyramids, enters the posterior fissure of the bulb and cord, and can be traced for some way along the back of the anterior column of the cord, into which it seems ultimately continued. Dr. A. observes, that it is contrary to our general impression that two portions of a nerve, supposed to have distinct functions, should be attached to the same point of the cerebro-spinal axis; and that the existence of a cord of communication with the anterior as well as the posterior column of the spinal cord removes any difficulty which might be created by the distinction in function between the two. *Dublin Journal, Nov.* 1836.

---

### *On the Mechanism of Bruit de Soufflet, or Bellows-Sound.* By D. J. Corrigan, M.D.

In February, 1829, Dr. Corrigan published in the Lancet an explanation of the manner in which this important sign is produced; and he now follows up the theory he advanced, and supplies its deficiencies. His account of the explanations which have been given, and his reasons for dissenting from them, we must pass over, and confine ourselves to his own theory, which is this :—In a natural condition, all the arteries are kept tensely distended by blood, and at each contraction of the heart the additional quantity of blood sent forward impels before it, "en masse," the blood already contained in the vessels; but, whenever the "bruit de soufflet," or its varieties, "bruit de rape, de lime," &c. are heard, this equable flow of blood and tense state of the vessels is interfered with, and a current-like or rippling motion of the blood is produced, which tends to excite vibrations in the vessels or arteries through which it passes; and, as the vessels are less tense, they are thrown more easily into vibrations by the irregular currents. Thus, a rippling motion of the blood, and a flaccid state of the vessels, are the mechanical conditions essential to the production of the sound. The circumstances under which the sound is heard are very various, and apparently contradictory: thus, it is heard in narrowing and in permanent patency of the cardiac orifices; in varicose aneurism; in the uterine vessels during pregnancy; in the heart and vessels after excessive loss of blood; momentarily in hysteria, &c., when the heart is sound; and it can be produced at will in any large artery, as the carotid or femoral, by pressing upon it to a certain degree with the finger or edge of the stethoscope. Dr. Corrigan endeavours to show that, in all these cases there is a rippling motion of the blood aud a flaccid state of the vessels, and therefore that his theory explains them all. Thus, when the femoral artery is pressed with the finger, it becomes comparatively flaccid beyond the narrowed portion, as the supply of blood is partially cut off, and therefore is insufficient to keep the artery tensely filled; and at the same time the pressure produces a rippling motion of the blood, by causing its particles to move with different degrees of velocity; those in the centre being impelled with the greatest rapidity, whilst those towards the sides are moved with varying velocities; currents are produced which impinge upon the flaccid sides of the artery, and cause corresponding vibrations. In aneurism of the thoracic aorta, with diseased aortic valves, the condition of the parts is well calculated to produce the sound; for the aneurismal sac is not kept distended, but becomes flaccid, from the imperfect aortic valves allowing a certain quantity of the blood expelled from the ventricle to pass back again. In tense aneurism there is pulsation, but no bruit de soufflet. Contraction of the auriculo-ventricular action of the heart is that lesion with which this sound is most frequently connected; for the blood rushes through the narrow orifice produced by the contracted state of the loose edge of the mitral valve into the relaxed and flaccid ventricle, and, both mechanical conditions being present, the sound is produced. In permanent patency of the mouths of the aorta, part of the blood, after each contraction, returns into the ventricle, and the aorta and its large branches become in some degree flaccid. In pregnancy, the bruit de soufflet is heard where the placenta is attached. The arteries of the placentar portion of the uterus are much enlarged, compared with the trunk that supplies them; and thus, in the intervals between the contractions of the heart, become somewhat flaccid, and in this condition receive the next dash of blood from the supply trunks: both conditions essential to the bruit de soufflet are therefore present. In varicose aneurism, a jet of blood passes into the vein at each pulsation, throwing the blood of the vein into irregular currents. After excessive loss of blood, the arteries are not completely filled with blood; it does not, consequently, move in its natural equable course, and this sound is produced. In hysteric and nervous persons, this sound is often heard for a short time, and is caused by irregular action of the heart and arteries producing an alteration in the motion of the blood, and a deviation from the natural tension of the arteries.

Dr. Corrigan illustrates his theory by several experiments. He fixed an elastic tube into an elevated vessel of water: whilst the water flowed through the pipe and equally distended it, no sound was produced; but, on pressing the tube with the finger, so as to diminish the supply of water beyond the point of pressure, the sides of the tube became flaccid, the fluid within was thrown into irregular currents, and a loud bruit de soufflet was produced. Bouillaud, in alluding to this experiment, attributed the sound to increased pressure on the fluid; but, to obviate this objection, the experiment was varied by attaching a smaller tube, of half the area, to the end of the first flexible tube: in the short time that the flexible tube was filling with water, its sides were flaccid, and the bruit de soufflet was heard; but, as soon as it was once filled, the water flowed through without any noise. The same effect was produced when a ligature was tied round the flexible tube, to diminish its caliber one-third. In both cases the sound was heard, and ceased to be heard, in exact accordance with the law laid down. Other experiments are given, but we must refer our readers to the excellent paper itself. *The Dublin Journal, November,* 1836.

---

*On the Vital or Self-moving Powers inherent in the Blood.* By R. M. Hawley, M.D.

At the meeting of the British Association held at Dublin, in 1835, Prof. Alison read a paper "On the Vital Properties of Arteries leading to Inflamed Parts," in which he attempted to prove by experiments that "a living or self-moving power is inherent in the blood itself, acquired by its purification in the lungs, and in some degree retained through its whole circuit." In the present short paper, Dr. Hawley adduces further evidence in support of these views, and considers that the following propositions are established by Dr. Alison's researches and his own:—1. That the motion of the blood from the branches of the pulmonary artery to the left auricle

*principally* depends, neither on the systole of the right ventricle, nor on the tenacity of the vessels, nor on the respiratory motions, but on a living self-moving power acquired by the blood, when renewed in the lungs. 2. That the same living power inherent in the blood is also a material cause of its motion through the capillaries of the aortic system. 3. That, in acute inflammation, this vital self-moving power of the blood is morbidly increased; while the tonicity of the capillaries of the inflamed part is diminished. 4. That some formidable diseases of the heart, lungs, and brain, usually considered primary, may be referrible to an altered state of this vital power of the blood; and perhaps oftener to its diminution than its augmentation.

*Edinburgh Journal for October*, 1836.

---

*Determination of the Question, which are the Nerves of Taste?* By B. ALCOCK, M.B.

THIS is a long and valuable paper, which was read at the meeting of the British Association, in 1836. It contains the results of many experiments made by the author, and critical remarks on the views of others. We regret that we must content ourselves with the bare enunciation of the inferences which the author has deduced from his experiments and reasonings, which are the following:—1st. That Taste is a special sensation. 2d. That it enjoys two media of perception. 3d. That its media of perception are the glosso-pharyngeal nerves and the lingual and palatine branches of the fifth nerves. 4th. That the glosso-pharyngeal nerves are not its special media. 5th. That the latter nerves both are sentient and influence muscular action; and 6th. That the spheno-palatine ganglion and chorda tympani have no influence upon either the existence or perception of the sense. *Dublin Journal, Nov.* 1836.

---

*On the Hemorrhagic Hepatization of the Lungs.* By R. KNOX, M.D., Lecturer on Anatomy in Edinburgh.

DR. KNOX's object in this short paper is to define more strictly the disease termed *pulmonary apoplexy* by Laennec, and to point out the true nature of the pathological state termed "the soft pulpy tubercle of the lungs" by Dr. Baillie. He wishes the term *pulmonary apoplexy* to be restricted to that state where there is an actual rupture of vessels, where there is "effusion of blood into the common cellular tissue of the lungs, and not merely the engorging of blood-vessels, or of the bronchial tubes." The affection described by Dr. Baillie, Dr. Knox believes to be simply this true pulmonary apoplexy, seen at some distance of time from the original attack. Dr. K. doubts whether Laennec ever witnessed a case of true pulmonary apoplexy, and suspects "that the pathological state which he had so designated was merely a hepatization caused by the gorging of the minute branches of the pulmonary artery with blood." This is what Dr. Knox terms *Hemorrhagic Hepatization*, and which he describes as follows, from a case recently witnessed by him: "The lung, being cut across, simply showed large portions of a deep red or even purplish colour, resembling masses of venous blood, and generally circumscribed. Notwithstanding the extreme resemblance these altered parts of the lung bore to apoplectic effusions, careful examination with a good glass totally disproved the fact of any extravasation. The tissues were hepatized, but unbroken, and much gorged with blood. From the vessels, when squeezed, fluid blood was forced out, even after the lapse of several days, and the whole appearance strikingly resembled, at first view, the *corpus cavernosum* of the penis. The smaller divisions of the bronchial tubes were filled with a gelatinous rose-coloured effusion; and this was present to whatever extent these tubes could be traced with a tolerably powerful magnifying glass; but this effusion was not present in the larger divisions of the bronchi. The minute subdivisions of the pulmonary artery were completely filled with a very dense coagulum, as far as it was possible to trace them, and their tunics showed numerous ossified specks. The pulmonary veins were empty, and quite round."

*Edinburgh Journal, October*, 1836.

